# Supplementary material for: Metabolomic and Transcriptomic Profiling Identified Significant Genes in Thymic Epithelial Tumor
Source: Metabolites. 2022 Jun 20;12(6):567. doi: 10.3390/metabo12060567 (PMC9228216; doi:10.3390/metabo12060567)
Supplement: Supplementary file 1 [file metabolites-12-00567-s001.zip › metabolites-1742929-Figures S1-S4.pdf]

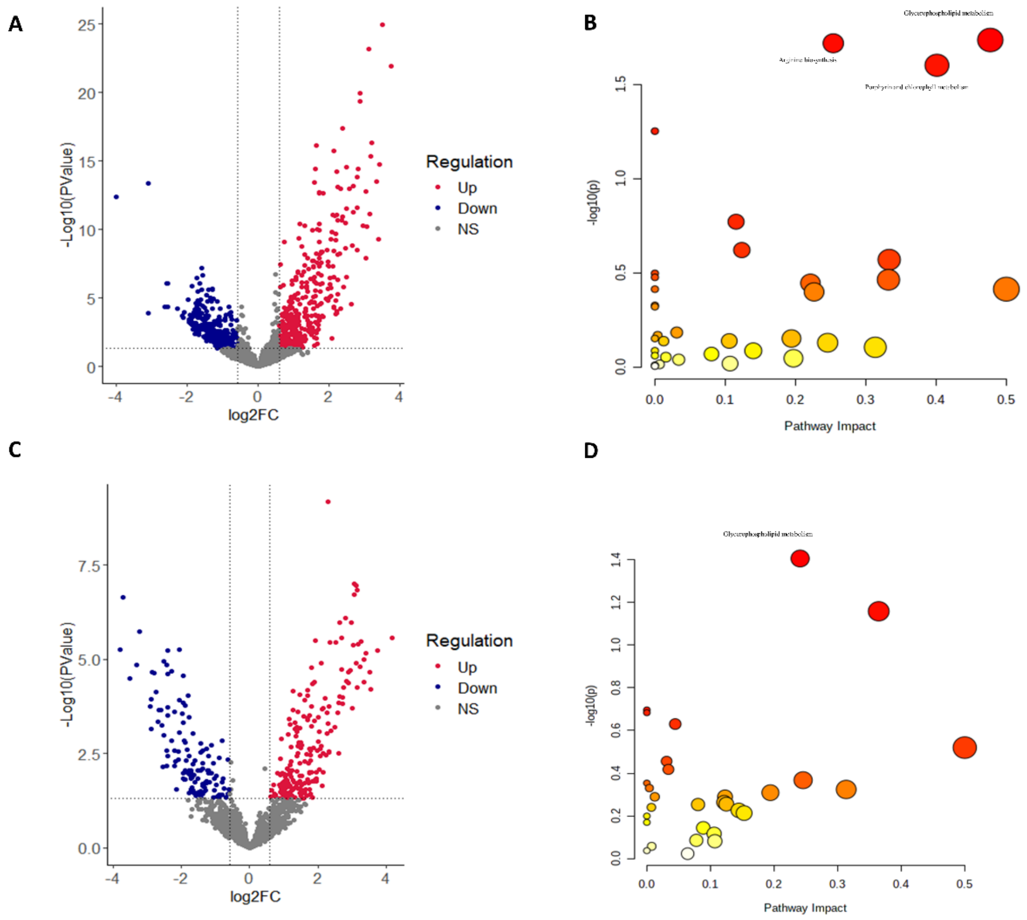

Figure S1. Volcano diagram revealing changes between the thymomas (A) or TCs (C) and ANTs; enrichment pathways of differential metabolic features between thymomas (B) or TCs (D) and ANTs (pathways with $p < 0.05$ were annotated)

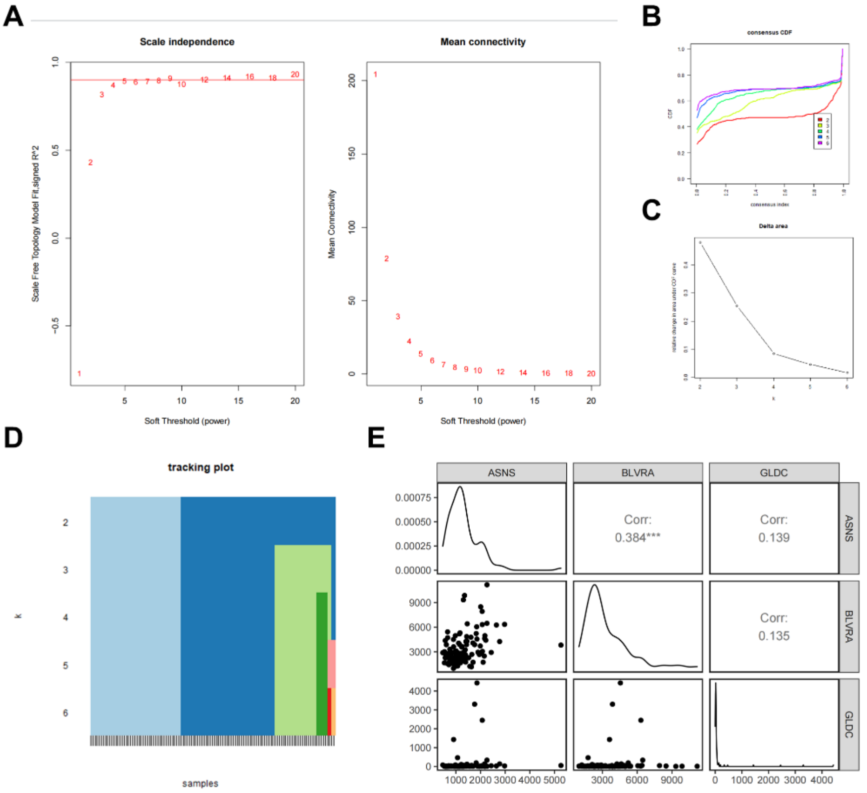

Figure S2. (A) A power of β = 4 (scale-free R2 = 0.87) was selected as the soft threshold in WGCNA; (B–D): The proportion of expression similarity and ambiguous clustering measures for the 137 TP-MRGs in consensus clustering; (E) Correlation analysis showed no collinear expression existed.

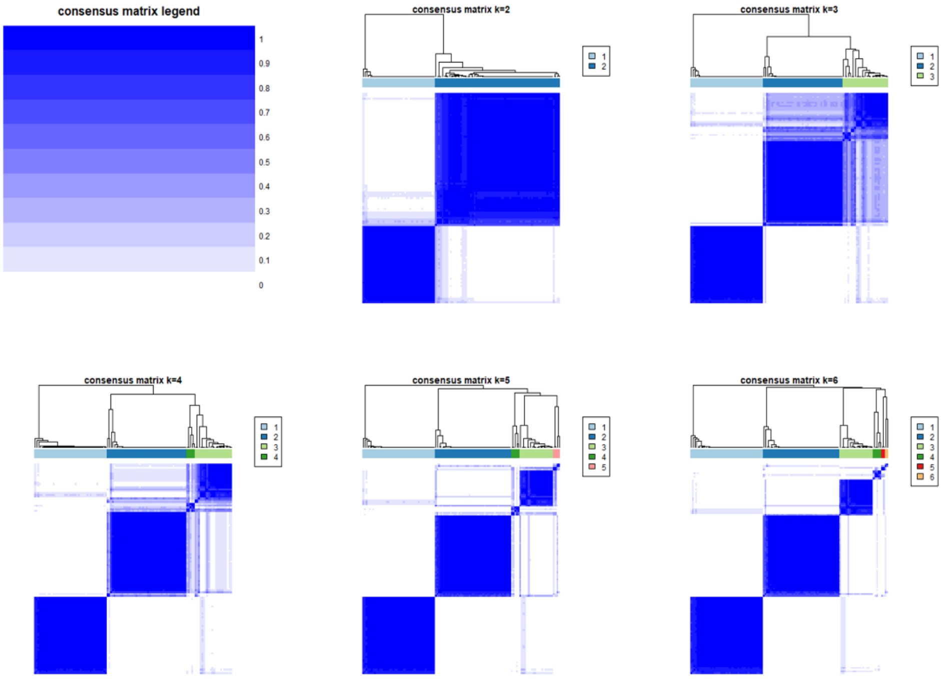

Figure S3. (A) The proportion of expression similarity and ambiguous clustering measures for the 137 TP-MRGs in consensus clustering.

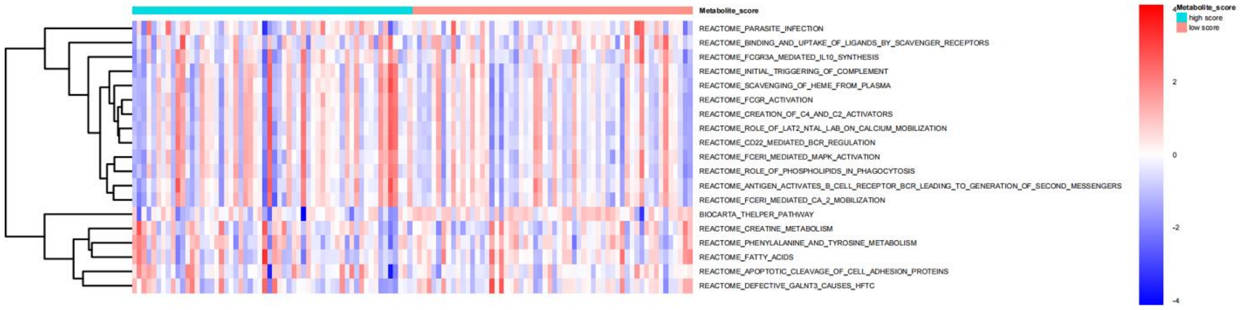

Figure S4. Gene set variation analysis (GSVA) showing the differentially enriched pathways in high- and low-metabolic-score groups.
