# Supplementary material for: Metabolomic and Transcriptomic Profiling Identified Significant Genes in Thymic Epithelial Tumor
Source: Metabolites. 2022 Jun 20;12(6):567. doi: 10.3390/metabo12060567 (PMC9228216; doi:10.3390/metabo12060567)
Supplement: Supplementary file 1 [file metabolites-12-00567-s001.zip › metabolites-1742929-Table S3.pdf]

Table S3. Multivariate analysis of the correlation of clinical variables with overall survival in TETs.

Clinical factors	Variables	P value	HR (95%CI)
WHO type	A/AB/B1 vs B2/B3/C	0.6	0.68 (0.15-3)
Masaoka stage	I/II vs III/IV	0.34	2.3 (0.41-13)
Sex	Male vs Female	0.7	1.4 (0.29-6.5)
Age	≤ 60 vs >60 (years old)	0.089	4.5 (0.8-25)
Cluster	Cluster1 vs Cluster2	0.55	1.7 (0.29-10)
Metabolite score	High score vs Low score	0.028	0.077 (0.0079-0.75)

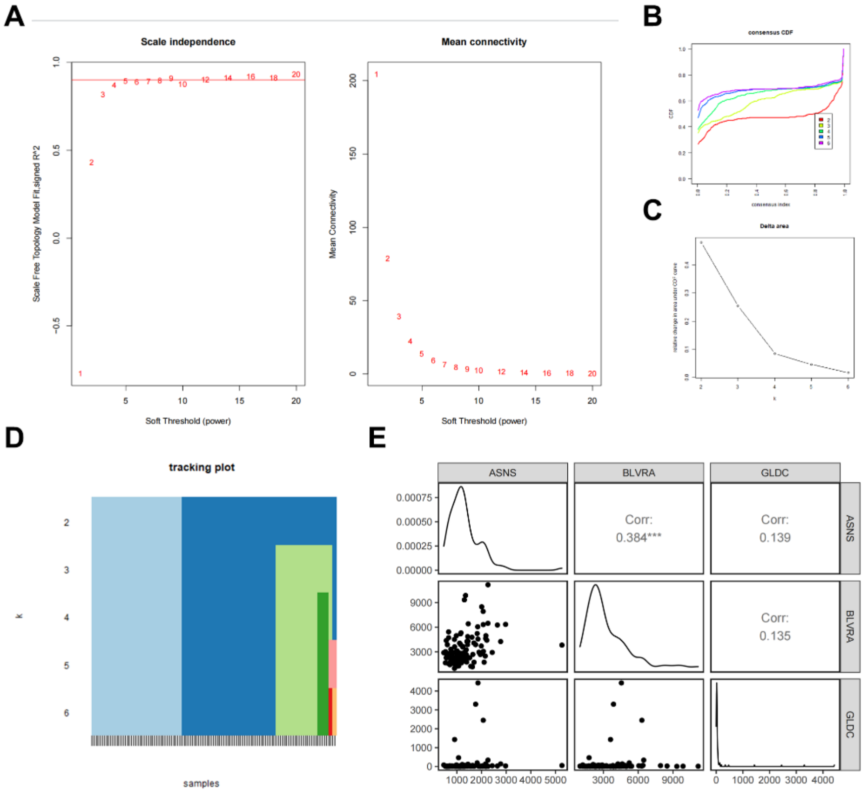

Figure S2. (A) A power of β = 4 (scale-free R2 = 0.87) was selected as the soft threshold in WGCNA; (B–D): The proportion of expression similarity and ambiguous clustering measures for the 137 TP-MRGs in consensus clustering; (E) Correlation analysis showed no collinear expression existed.

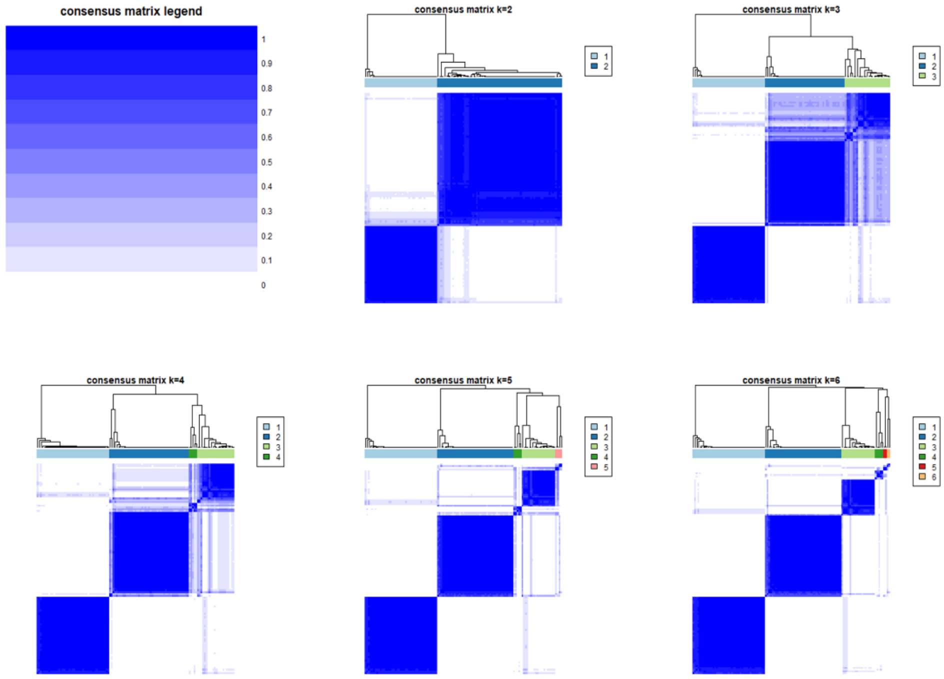

Figure S3. (A) The proportion of expression similarity and ambiguous clustering measures for the 137 TP-MRGs in consensus clustering.

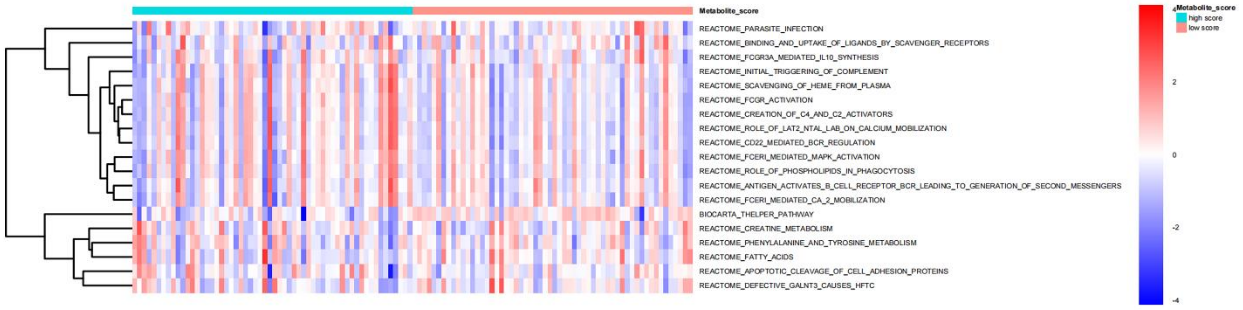

Figure S4. Gene set variation analysis (GSVA) showing the differentially enriched pathways in high- and low-metabolic-score groups.
